# An integrative data analysis platform for gene set analysis and knowledge discovery in a data warehouse framework

**DOI:** 10.1093/database/baw009

**Published:** 2016-03-17

**Authors:** Yi-An Chen, Lokesh P. Tripathi, Kenji Mizuguchi

**Affiliations:** National Institutes of Biomedical Innovation, Health and Nutrition, 7-6-8 Saito-Asagi, Ibaraki, Osaka 567-0085, Japan

## Abstract

Data analysis is one of the most critical and challenging steps in drug discovery and disease biology. A user-friendly resource to visualize and analyse high-throughput data provides a powerful medium for both experimental and computational biologists to understand vastly different biological data types and obtain a concise, simplified and meaningful output for better knowledge discovery. We have previously developed TargetMine, an integrated data warehouse optimized for target prioritization. Here we describe how upgraded and newly modelled data types in TargetMine can now survey the wider biological and chemical data space, relevant to drug discovery and development. To enhance the scope of TargetMine from target prioritization to broad-based knowledge discovery, we have also developed a new auxiliary toolkit to assist with data analysis and visualization in TargetMine. This toolkit features interactive data analysis tools to query and analyse the biological data compiled within the TargetMine data warehouse. The enhanced system enables users to discover new hypotheses interactively by performing complicated searches with no programming and obtaining the results in an easy to comprehend output format.

**Database URL:** http://targetmine.mizuguchilab.org

## Introduction

The proliferation of high-throughput ‘omics’ experiments has led to a surge in the availability of biomedical data that need to be properly analysed. Leveraging biological information from different data types yields deeper insights into gene function and provides a better understanding of the biological process under study, which can be further transformed into actionable research. For instance, drug repositioning (i.e. new uses for existing drugs) (1) and combinatorial drug treatments (2) necessitate a systems-level mapping of drug–target interactions and their influence on cellular networks. Cellular networks themselves comprise multiple organizational layers made up of different types of biomolecular interactions such as microRNA (miRNA)–target interactions (MTIs), protein–protein interactions (PPIs) and transcription factor (TF)–target gene interactions, which together modulate the functioning of the living systems. The ability to correlate these data with gene expression patterns and *a priori* knowledge of the genetic determinants of various diseases is key to a deeper understanding of disease mechanisms and development of better therapeutic strategies.

However, integration of the vast and scattered array of biological information is a multifarious scientific challenge, for reasons ranging from inconsistencies in data gathering to heterogeneous and often incompatible formats used to store and manage biological data in different repositories. Despite these obstacles, the immense benefits of an integrative approach in disease biology and drug discovery have spawned numerous efforts to develop different types of frameworks and tools for integrating diverse biological data types (3–10) and for functional analysis of large gene sets. Gene set functional enrichment relies on a statistical analysis of the relative abundance of biological themes associated with a given gene list and identifies themes (and associated genes) that are overrepresented and therefore, likely to be more relevant to the biological conditions under study. For instance, the DAVID gene functional classification resource employs a heuristic approach to grouping genes into modules based on similarities in the biological annotations and provides a set of tools for functional analysis of user-supplied gene lists (11). On the other hand, Enrichr employs pre-defined gene set libraries to assist functional enrichment analysis of large gene lists (12). However, most of the available tools that facilitate gene set functional enrichment provide a standalone web interface and have not been integrated into a more general data-mining platform; such a platform is often crucial to refine and validate gene set functional enrichment results and for further characterization of gene sets in drug discovery and related applications.

We have previously developed TargetMine, an integrated data warehouse based on the versatile InterMine framework (8, 10, 13), which models biological entities (such as genes and proteins) as ‘objects’ and their relationships as ‘references’ to other objects. TargetMine was optimized for target discovery and prioritization of candidate genes (14) and was successfully employed for gene prioritization in various studies including hepatitis C virus pathogenesis (15–17), carcinogen-induced lung tumourigenesis (18), Alzheimer’s disease (19) and acute myeloid leukaemia (20).

However, TargetMine is primarily a data warehouse; while it allows the users to obtain quickly which of the predefined biological themes are enriched within the user-supplied gene (and protein) lists, it provides limited scope to combine sophisticated analytical and visualization tools or to define a data analysis workflow. For example, given a set of genes, multiple queries are necessary to retrieve the different interaction types that these genes engage in. Then to obtain a combined network, one has to merge the output files containing the different interaction types manually and use standalone network graphics tools such as Cytoscape (21).

**Figure 1:**
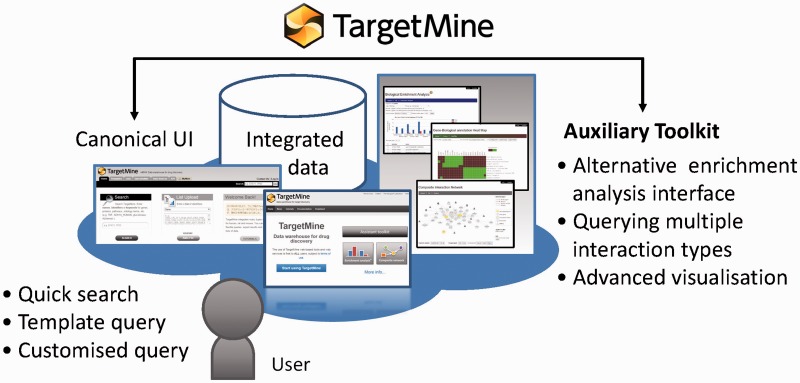
TargetMine enables the users to discover new hypotheses interactively, by performing complicated searches without any scripting and programming efforts on the part of the user and also by obtaining the results in an easy to comprehend output format.

Here we describe significant new developments of the TargetMine system, including newer datatypes and a new auxiliary toolkit to assist with data analysis and visualization and to address the limitations in TargetMine. To retain the advantage of being a gene set analysis tool built into a data warehouse system, we decided to utilize the Application Programming Interfaces (APIs) provided by the InterMine framework so that advanced analysis and visualization programmes can be combined with TargetMine (Figure 1). At the same time, we aimed to transform TargetMine from a target discovery tool to a unified integrative data analysis platform for assisting drug discovery and development more generally. To facilitate this transition, we have embarked upon two key objectives: (i) to identify and include new suitable data sources and more effective approaches to inter-connecting them and (ii) to introduce an enhanced visualization and workflow-like capabilities to facilitate better data sharing and analysis. We will discuss these developments and demonstrate the effectiveness of these new features using case studies.

## Inclusion of New and Embellished Data Sources and Data Models in TargetMine Have Provided a More Expansive Coverage of the Biological Data Space

To cover the wider biological data space and assist drug discovery and related research, we have focused on biological data associated with three major areas—drug–target interactions, gene–disease associations and biological mechanisms. Below, we will discuss how these different data types have been modelled and enabled new analysis capabilities within TargetMine below.

### Drug–target interactions

Protein–chemical compound interactions (PCIs) are scattered across different databases that provide different types of information; moreover, the objects (chemicals) from different data sources are not fully unified. To overcome these limitations, we have gathered PCI data from ChEMBL (22), DrugBank (23), Protein Data Bank [PDB; www.wwpdb.org (24)] and PubChem BioAssay (25). We have then unified the chemical compounds from these repositories by using the InChI system developed by the International Union of Pure and Applied Chemistry (IUPAC) (see Materials and Methods for details) to compile an exhaustive collection of PCIs combining the drug–target interactions in DrugBank with PCIs in ChEMBL and PubChem BioAssay and protein structures that contain heterogen groups in PDB.

**Figure 2:**
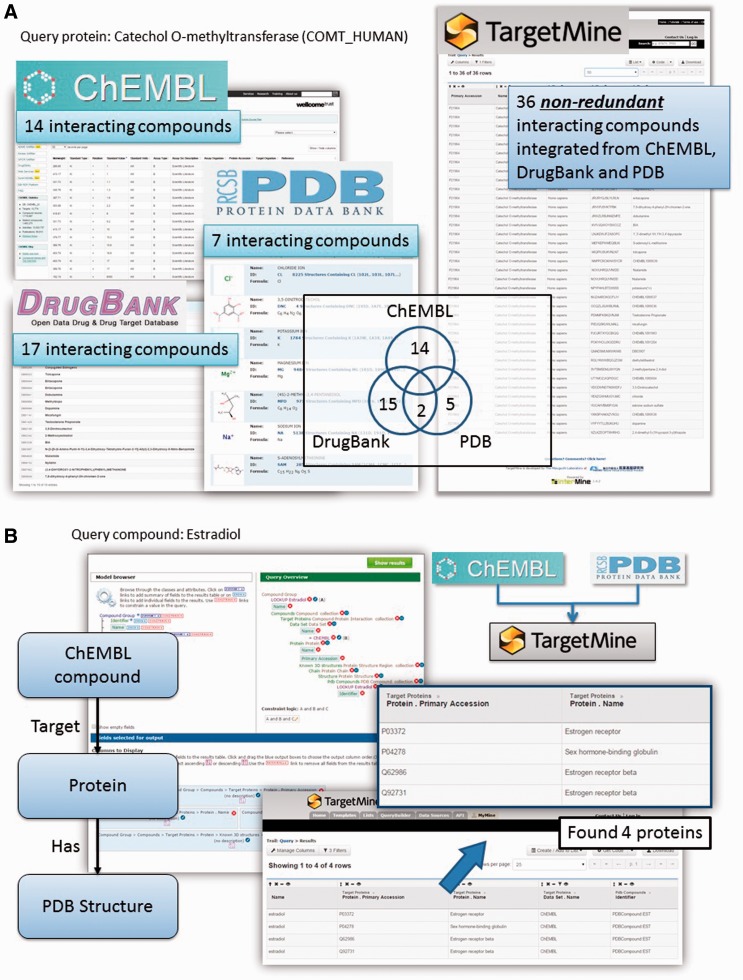
Querying drug–target interactions (DTIs) with TargetMine. (**A**) TargetMine provides a better coverage of PCIs by using a unified PCI repository than each of the individual data sources. Results are shown for a query ‘Given a protein, find all the compounds that target this protein’, with the protein catechol *O*-methyltransferase as an example. (**B**) A query of ‘Given a drug, identify all the targets (in the ChEMBL database) that have been co-crystallized with this drug (in PDB)’ with oestradiol identified four known targets complexed with this drug.

This integration allows the users to connect DrugBank and ChEMBL drugs to complex structures in PDB using the list function and the query builder tool in TargetMine (see Figure 2A and B for query examples). Our implementation also permits the users to identify chemicals and drugs that interact with a given set of genes/proteins and integrate them into gene–disease associations and regulatory networks (see below for specific examples). Such an analysis will help assess the potential impact of various drug treatments on cellular functions.

### Gene–disease associations

A better understanding of links between genes, pathways and specific diseases is likely to contribute to the identification of newer and better drug targets. However, explicit gene–disease associations are difficult to obtain in general. Genome-wide association studies (GWAS) that seek to associate single nucleotide polymorphisms (SNPs) with specific phenotypes and/or diseases within a human population have uncovered scores of genetic variants associated with complex disease traits (26–28). Therefore, we obtained the GWAS data from National Human Genome Research Institute (NHGRI) and matched them carefully against Disease Ontology (DO) terms (see Materials and Methods) to generate a set of uniformly annotated gene–disease relationships.

This integration enabled the users to perform queries such as ‘Given a disease name or a disease identifier (DO), find the related SNPs and genes from any relevant genome-wide association study and identify the drugs that are known to be associated with these genes’.

### Mechanisms of gene and protein function in biological systems

To elucidate mechanisms of gene and protein function more effectively, we have first included different types of biomolecular interactions (MTIs, PPIs and TF-target gene interactions) that correspond to different regulatory levels in the cellular networks but are scattered across various databases and are thus hard to obtain from other integrated resources in the form of a unified repository. Second, we have included simplified (and unified) biological themes that assign easy-to-interpret functional roles to various genes and proteins. Third, we have included cell/tissue-specific gene expression patterns from the Gene Expression Barcode database (hereafter referred to as Barcode) (29) to enable comparisons between gene expression levels between different cell and tissue types.

#### MTIs, PPIs and TF-target gene interactions

**Figure 3:**
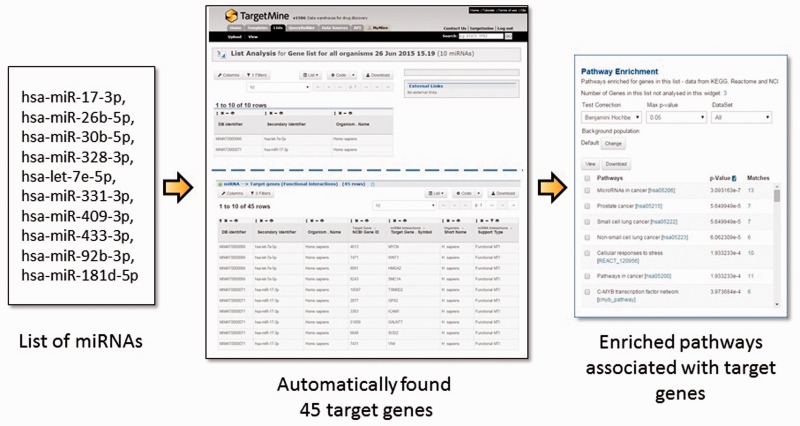
The users may upload a list of miRNAs, generate a list of their targets and process the target genes for functional enrichment analysis using KEGG pathways. In the given example, a list of 10 miRNA IDs were uploaded and by using a query template of ‘Given a list of miRNAs, retrieve all their target genes’, a list of 45 target genes were created and their enriched pathways identified immediately. Alternatively, one can start from a list of genes and use a query template of ‘Given a list of genes, retrieve all the miRNAs which target the genes within the list’.

Post-transcriptional gene regulation by miRNAs is widespread phenomenon that influences a broad spectrum of cellular processes in metabolism, development and diseases (30–34). To obtain a systems level mapping of the miRNA ‘targetome’, that is, specific MTIs and their emergent behaviour, we have included miRNA information from miRBase (35) and MTIs from miRTarBase (36, 37) (see Materials and Methods). This integration has enabled the users to infer comprehensive MTI networks from either a list of miRNAs or that of genes, and connect immediately to related annotations such as enriched pathways (see Figure 3 for query examples).

PPI data provide valuable insights into cellular processes and their involvement in various diseases. As most PPI databases differ in scope and content, a combined repository is necessary to obtain a better coverage of protein interactomes (38). Therefore, we included in TargetMine PPIs from BioGrid (39), iRefindex (40) and a comprehensive study (41) that defined high-quality binary interaction datasets.

An expansive PPI repository may still be beset with noise and false positives and thus, we performed a confidence assessment of the combined PPI repository and defined a reliable high-quality subset termed ‘high-confidence direct physical PPIs’ (see Materials and Methods). These interactions were further analysed to infer the network topological properties (42, 43). We classified genes/proteins as network ‘hubs’ that are likely to have a higher ability to influence biological networks via multiple PPIs and/or ‘bottlenecks’ that regulate the flow of signalling information and therefore represent central points for communication in an interaction network (43) (see Materials and Methods). The PPI data integration has enabled the users to query the interacting partners of a gene/protein or a list of genes/proteins of interest and to infer overall PPI networks involving these genes/proteins. The users may also query for the degree and betweenness coefficients for a list of genes/proteins and identify hubs and bottlenecks among them.

TF-target gene interactions determine gene expression patterns that modulate key cellular functions and some TFs such as nuclear receptors are important drug targets (44). Given their considerable significance, we included expert-curated experimentally validated human TF-target gene interaction data from AMADEUS (45), OregAnno (46) and HTRIdb (47) to create a combined repository in TargetMine (see Materials and Methods).

The inclusion of different interaction types in TargetMine enables users to perform complicated queries, not only for a single interaction type (e.g., ‘Given a list of genes, retrieve all the upstream and/or downstream TF-target relations observed within the list’) but also across multiple network types. For instance, the users may upload a list of (e.g., differentially expressed) miRNAs, generate a list of their targets and identify hubs and bottlenecks among them. The users may further process the target gene sets to query for compounds (or specifically drugs) that are known to target them and to perform functional enrichment analysis (see below).

#### Enhanced functional annotation with pathways, biological themes and orthologues

To obtain a comprehensive mapping of functional associations of gene products, we incorporated pathway data and gene–pathway associations from the KEGG (48), Reactome (49) and NCI-Nature curated PID (50) databases and gene–function associations from the Gene Ontology (GO) consortium (51) within TargetMine. Collectively, they have provided a vast repository of functional annotations for human, mouse and rat genes.

**Figure 4:**
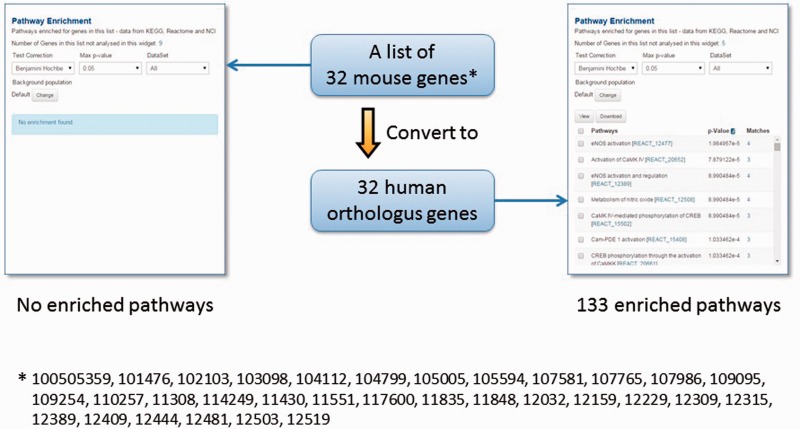
The orthologue conversion system in TargetMine can help circumvent the lack of functional annotations. In this example, the original list of 32 mouse genes produced no enriched pathways (with the default cut-off of statistical significance). By converting this list to 32 human orthologous genes, 133 enriched pathways were identified.

However, genome annotations are far from complete and even the existing functional annotations are biased towards specific biological processes, chiefly due to the differences in experimental systems that have been employed to investigate different model organisms (52). Therefore, we have included protein sequence and structural domain assignments from the InterPro (53) and Gene3D (54) repositories to expand the repertoire of functional annotations in TargetMine. We have also mapped orthologous gene associations across human, mouse and rat genomes (see Materials and Methods), as orthologous genes (that are descended from a common ancestral gene as a consequence of speciation) are believed to retain similarity in structure and function (55–58). The one-click gene conversion function within the lists page of TargetMine allows the users to circumvent the limited species-specific functional annotations (see Figure 4 as an example; from our experience, we often encounter similar real-life situations).

On the other hand, gene set functional enrichment analysis may often generate a large number of enriched pathways/GO terms, making it difficult to capture meaningful biological themes. To facilitate a relatively quicker gene set analysis with a manageable and meaningful approximation of biological themes associated with query gene lists, we included two additional data sources that provide a broader and simplified overview of gene function, Integrated Pathway Clusters (IPCs) and GO Slims.

We have previously constructed IPCs based on gene set overlap across pathways from KEGG, Reactome and NCI-PID and shown that IPCs have provided a convenient way to identify broad functional categories relevant to the biological phenomenon under study (59). GO Slims are selected subsets of GO terms that provide a broader overview of GO and GO annotated gene products. Thus, the inclusions of these two resources have enabled the users to obtain swiftly a relatively simplified characterization of gene products.

#### Cell and tissue-specificity of gene expression

Spatiotemporal gene expression plays a key role in regulating gene function and also contributes to the diversity and functions of various cells and tissue types. Therefore, we retrieved the data from Barcode (29) and obtained gene expression profiles of individual genes in specific cells and tissues (see Materials and Methods).

This implementation allowed the users to compare gene expression between different cells and tissues and identify genes with high and/or minimal expression in select cell types. The users may also compare the gene expression profiles of specific human, mouse and rat genes and further refine their analyses and hypotheses. For instance, users can potentially refine biomolecular networks by retaining only those genes that are ‘sufficiently’ expressed in specific cells and tissues. The implementation also allows users to perform queries such as ‘Given a list of compounds (or drugs), identify target hubs and bottlenecks that are of known protein structure and that are highly expressed in specific cell/tissue types’.

## Improved Data Analysis and Visualization Workflow with TargetMine

We have developed an auxiliary toolkit to assist with data analysis and visualization in TargetMine. This implementation was aimed at establishing a data analysis platform without any scripting and/or programming efforts on the part of the user (Figure 1).

**Figure 5:**
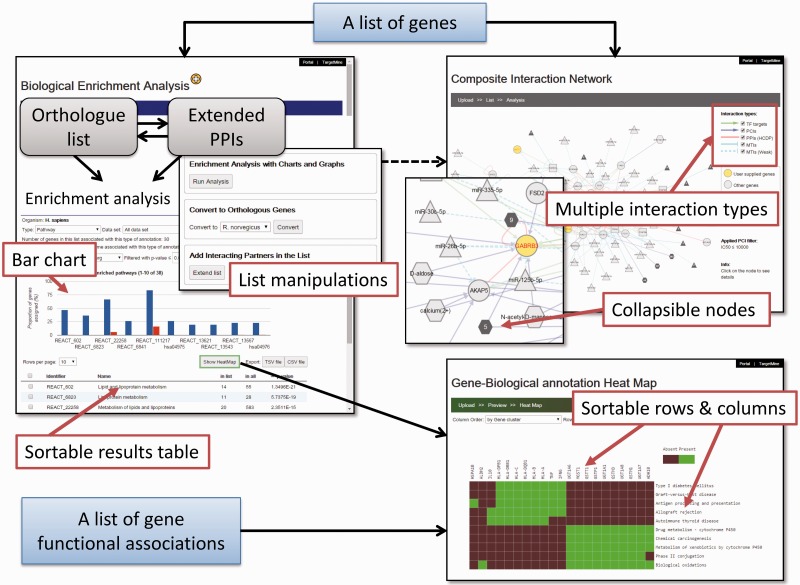
A screenshot of the TargetMine auxiliary toolkit for data analysis and visualization.

The auxiliary toolkit enhances TargetMine currently in two ways: (i) biological enrichment analysis with automatic data conversion, graphs and charts; and (ii) composite interaction network (Figure 5). These functions can be accessed from List pages in TargetMine or via designated input forms. After the auxiliary toolkit analysis, the user can go back to gene or pathway summary pages in TargetMine for further examination. The analysis results are exportable in tab/comma-separated text format and in high-resolution graphic images for incorporation into publications and presentations.

### 

#### Biological enrichment analysis

The enhanced functionality can be accessed by following the ‘Enrichment+’ link on the gene ‘List Analysis’ page in TargetMine or using a newly designed upload form. The users can upload a list of genes, extend the query list by including PPI partners and perform orthologue conversion for subsequent enrichment analysis, with only a few clicks of the mouse (Figure 5); these processes would involve multiple steps invoking several queries in the original List form. The enrichment analysis results are returned as a table and a histogram that display the distribution of the enriched biological themes in the query list *vis-à-vis* the background (whole-genome annotations). The user may sort the output table (and the histogram) by *P* values or the number of annotated genes (foreground or background) or the biological theme names (Figure 5). In the case of KEGG pathway enrichment analysis, the user may click on the pathway name in the output table and the associated genes are automatically sent to the KEGG website and highlighted on the corresponding pathway map.

A global perspective on the functional proximity and similarity between different genes in the query list (that correspond to the biological process under study) rapidly facilitates the selection of biologically relevant gene signatures and eventually, biomarker discovery. To assist such processes, we have introduced an association heatmap function. Clicking the ‘Show HeatMap’ tab will enable the users to view the selected gene-biological theme annotations as a heatmap. There, the associations are visualized as a mosaic such that genes that share a high proportion of functional attributes were clustered together and thus appeared as bright spots on the grid (see Materials and Methods). The association heatmap is interactive; when the user hovers the mouse over a cell, a white tooltip appears to display the gene and biological theme corresponding to that cell. The user may customize the output by sorting the grid by either the gene name or the biological themes; the users may also customize the display colours for the cells of the grid. In addition to viewing the enrichment analysis results, the users can also upload their own (arbitrary) gene-biological theme association tables (such as those exported from TargetMine) or matrices (in a tab-delimited format; see the example available in the heatmap interface), transform it into a heatmap and sort the rows/columns (Figure 5). The implementation also allows the heatmap to be exported as a publication quality image and the underlying matrix to be downloaded into excel sheets.

#### Composite interaction networks featuring multiple interaction types

**Figure 6:**
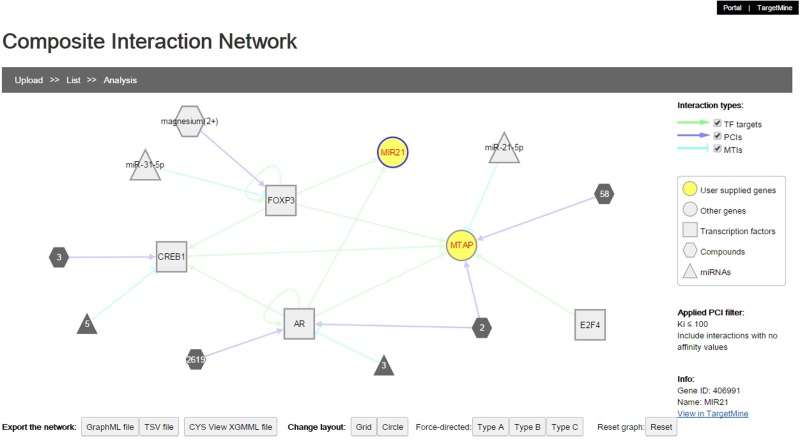
The users can infer composite interaction networks that include different types of biomolecular interaction types such as PCIs, MTIs, PPIs and TF–target gene interactions and identify new regulatory associations brought about by interactions between different operational levels of the cellular networks.

We implemented a network visualization function. Users can supply a list of genes to construct and visualize a composite interaction network that includes all the biomolecular interactions within TargetMine, i.e. PPIs, MTIs, PCIs/drug–target interactions and TF–target gene interactions, which are associated with the query genes. In a composite interaction network, the different biomolecular interaction types are illustrated in different symbols and colours and the users can choose a variety of layouts (such as force-directed, circular and organic) and also display/undisplay-specific interaction types. The users may also filter PCIs (ChEMBL only) by selecting a desired activity threshold (Figure 6). Achieving the same output using TargetMine would necessitate making multiple queries to retrieve the different interaction types, merging the output files manually and employing standalone network graphics tools such as Cytoscape (21) to visualize a combined network.

The implementation of the auxiliary toolkit achieved the new functionality without altering the core portion of the InterMine code base, while maintaining the unified interface as much as possible. This modular design allows great flexibility for future developments. The current functions are sufficient for the intended analysis (e.g. examining associations between genes and biological themes as a mosaic) but as needs arise, we can easily adopt new and more sophisticated libraries for data analysis and visualization.

## Demonstration of the Usefulness of TargetMine as an Enhanced Knowledge Discovery Tool—A Case Study

**Figure 7:**
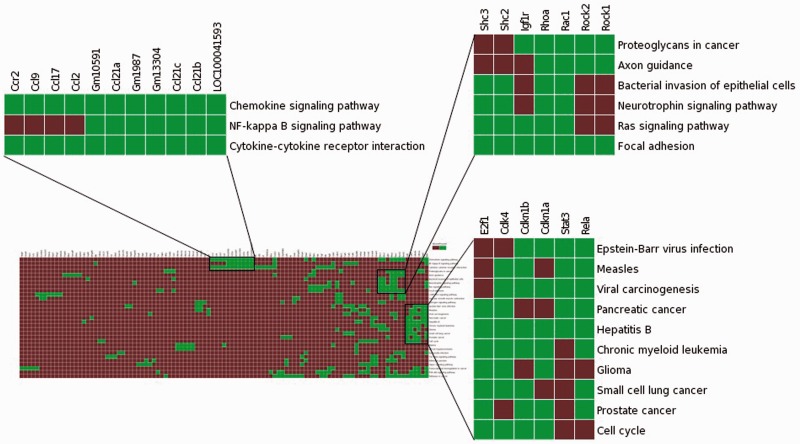
Visualization of the enrichment analysis of gene–functional associations of genes upregulated in lung tumourigenesis (in Stat3 knockout mice) using the heatmap function highlighted subsets of functionally related genes and suggested plausible links between Ccr2, NF-kappa B signalling and enhanced inflammatory responses in carcinogen-induced lung tumourigenesis.

To assess the effectiveness of enhanced TargetMine in knowledge discovery, we performed a functional analysis of genes involved in modulating the role of the TF Stat3 in carcinogen-induced lung tumourigenesis in mice. We begin by uploading a list of genes (Table S1) that corresponded to a PPI network constructed from genes that were upregulated in Stat3 knockout mice (18) to the ‘biological enrichment analysis’ portal menu. We then visualize the output from the KEGG pathway enrichment analysis using the heatmap function (Figure 7). We see immediately that the deletion of Stat3 led to a significant increase in the genes associated with chemokine production (Ccl2, Ccl9, Ccl17, Ccr2 and Ccl21c), as they were mapped to the enriched KEGG pathway ‘Chemokine signalling pathway’. This observation is consistent with previous reports and supported the hypothesis that Stat3 deletion actively contributes to enhanced chemokine production in lung tumour cells, thereby contributing to enhanced inflammatory responses (18).

The association heatmap function is also useful for providing potentially novel insights into this process. In this example, the heatmap reveals distinct clusters of functionally related genes, one of which includes genes mapped to enriched KEGG pathways ‘Chemokine signalling pathway’, ‘Cytokine–cytokine receptor interaction’ and ‘NF-kappa B signalling pathway’ (Figure 7). This appears to be consistent with previous observations that NF-κB activates a number of cytokines and chemokines in lung inflammation (60). It is also known that mice deficient in Ccr2 gene is unable to activate NF-kappa B signalling in pulmonary infection (60). Taken together, Ccr2 upregulation may contribute to increased NF-kappa B signalling leading to enhanced chemokine production, which in turn significantly contributes to increased inflammatory responses in early stage carcinogen-induced lung tumourigenesis in Stat3-deficient mice. Such insights would not be easily achieved using the standalone pathway enrichment analysis tools such as enrichment widgets within TargetMine.

## Comparisons with Similar Resources

As a data warehouse, TargetMine is not an alternative to large public databases, rather it is designed to provide an alternative usage to collate and summarize biological information for large gene sets in a quick, effective and simplified manner. To enhance the TargetMine user experience, we have designed an auxiliary toolkit to provide visualization and workflow-like functions within a data warehouse framework with no programming efforts on the part of the user. Collectively, our system facilitates rapid and efficient biological data gathering and integrated data analysis and summary in a single user-friendly interface.

Although some publicly available resources such as the DAVID gene functional classification tool (61, 62), Enrichr (12), The Comparative Toxicogenomics database (a curated database of chemical–gene/protein interactions, chemical–disease and gene–disease relationships) (63) and commercial resources such as Ingenuity® (Redwood City, CA) and MetaCore^TM^ (GeneGo, St. Joseph, MI) may offer additional data types and more specialized tools for statistical data analysis and visualization, they are not integrated into a data warehouse system. In contrast, TargetMine offers higher flexibility and readily customisable data types. For instance, the users may upload their own sets of gene annotations for analysis using the heatmap tool. We have designed our own data models for annotations such as unified chemical entities, gene–disease associations, high-confidence PPIs, hubs, bottlenecks, orthologue conversion and gene expression profiles. These annotations were unique and highly integrated within TargetMine and therefore many of our query examples are unlikely to be available from other comparable tools (at least at this level of easiness).

## Conclusions and Future Developments

The expanded repertoire of data types in TargetMine has significantly enhanced the coverage of the biological data space. We have developed an auxiliary toolkit to assist TargetMine users to visualize and analyse the diverse biological datatypes seamlessly. The enhanced system enables the users to perform complicated queries, select and define query parameters and engage multiple datasets simultaneously, thereby providing a wide range of possibilities for the users to interrogate the biological data space that may not be easily achieved using other tools. It also allows the users to export publication quality images and the results in a variety of formats such as Excel sheets.

The enhanced system is structured to accommodate increasingly available biological data from large-scale experiments and to adopt newer and more sophisticated tools to visualize and analyse such data. TargetMine is regularly updated (at least once a month) to provide up-to-date functional annotations. We will continue to enhance the repertoire of functional associations in TargetMine by inclusion of new data types such as biochemical reactions in the context of PPIs and further regulatory relationships and enhanced coverage of gene–disease and gene–drug associations. We also aim to complement the enhanced data types in TargetMine with more sophisticated and user-friendly features for data analysis and visualization. For instance, we plan to extend the newly designed analysis functionality, which presently accepts only genes as input, to accept other identifiers such as proteins, probesets and chemical compounds in the near future. We aim to enhance the heatmap interface with additional features such as more sophisticated clustering algorithms, inclusion of dendrograms and automated identification and extraction of gene clusters. We also aim to enhance the comprehensive interaction network by including the visualization of directed interactions and mapping of functional associations and topological attributes to highlight node specificities. Furthermore, we also aim to introduce a workflow function for target prioritization and further investigation. These features are likely to enhance the ability of TargetMine to aid drug discovery and related research.

## Materials and Methods

### New data models and parsers

New data models and parsers were written in Java programming language and subsequently integrated into the InterMine/TargetMine source code.

### Drug (chemical compound)–target interactions

Chemical compounds from different data sources were integrated by using the InChI system. InChI stands for International Chemical Identifier, a string that represents the exact stereochemistry of a compound, generated by a computer algorithm. InChIKey is a shorter hash key version of InChI, a string with fixed length. The InChIKey is a 27-character code consisting of three blocks separated by hyphens. The first block consists of 14 characters and encodes the molecular skeleton. The InChI system is the only standard structure representation in the public domain, and available in all the chemical resources that we integrate in TargetMine. We collated the compounds that share the same InChIKey of the first 14 characters in a CompoundGroup object, and associated this object with the Protein class via two newly designed classes, Compound and CompoundProteinInteraction. To store the experimental assays, we introduced the CompoundProteinInteractionAssay and Activity classes that compiled the PCIs documented in ChEMBL. We selected from ChEMBL only those protein–compound interactions that were qualified with confidence_score ≥4, assay_type = ‘B’ (BINDING), target_type= ‘SINGLE PROTEIN’ and at least one of the standard_values (‘IC50’, ‘Kd’ and ‘Ki’) =  ‘≤10 000 nM’.

### Gene–Disease associations

GWAS data were retrieved from NHGRI and the GWAS study tags for individual genes were mapped to DO terms using a manual approach based on exact or closest key word matches. Next, we defined two new classes, the SNP class, which was referenced to the gene class and the GenomeWideAssociation class, which was referenced to the SNP and the DOTerm classes.

### MTIs, PPIs and TF–Target Gene Interactions

#### miRNA

We designed MiRNA and MiRNAPrimaryTranscript classes to collate miRNA information from miRBase (35). We also introduced three additional classes: MiRNAInteraction for storing the experimentally validated MTIs, MiRNAEvidence for MTI classification as ‘weak’ or ‘non-functional’ and MiRNAExperiment for the experiments that were used to characterize MTIs, as defined in miRTarBase (36, 37).

#### PPIs

For convenience, all PPIs in TargetMine were stored as gene–gene interactions. PPIs that were supported by at least two different experimental methods or two independent publications were judged to be high-confidence PPIs. High-confidence direct physical PPIs were defined as a subset of the high-confidence PPIs that satisfied at least one of the following conditions: (i) annotated as direct physical interactions in the source repositories, (ii) identified by at least one qualified experimental procedure [such as yeast two-hybrid assays, fluorescence energy resonance transfer, atomic force microscopy and other methods that can detect direct physical interactions (64)], and (iii) the supporting PMIDs. Only the largest singly connected component of the high-confidence direct physical PPI network was considered for the topological analysis. Network topological properties such as ‘node degree distribution’ and ‘betweenness’ measures were computed using the Java Universal Network/Graph Framework (JUNG) library (http://jung.sourceforge.net/). ‘Hubs’ and ‘bottlenecks’ were defined as the top 10% of the nodes ranked by ‘node degree distribution’ and ‘betweenness’ measures, respectively.

#### TF–target gene interactions

We modified the existing InterMine interaction model to define a new class, ProteinDNAInteraction to store TF–target gene interactions; TFs were labelled ‘source’ and the target genes ‘targets’. We further introduced the BindingSite class to reflect the TF binding sites in the regulatory regions of the target genes. All the entries were manually processed to unify gene identifiers and remove redundancies.

### Pathways and biological mechanisms

#### Pathways

We created new parsers to retrieve and collate gene–pathway associations from the three databases into an expanded, customized and refined ‘Pathway’ class that was described previously (14).

#### GO

To store and manage GO annotations, we employed the default InterMine GOAnnotation class but with custom data parsers. The GO terms were initially assigned to proteins and then transferred to genes post-data integration procedure.

#### Protein sequence and structural domains

We created new parsers to retrieve and collate protein sequence domain annotations from InterPro into the ProteinDomainRegion class that was described previously (14) and protein structural domain assignments from Gene3D into the newly defined StructuralDomainRegion class. Both these classes were referenced to the Protein class.

#### Orthologous relationships

Human, mouse and rat protein sequences were scanned against each other by using SSEARCH (65) with a threshold of *e*-value ≤ 10^−3^ and the mutual best hits were defined as orthologues. To store the orthologous relationships, a new attribute ‘orthologProteins’ was included within the Protein class.

#### IPCs

To store and manage the IPCs, we introduced the new class IntegratedPathwayCluster and referenced them to the Pathway class (59).

### GO slim

We extended the default InterMine GOAnnotation class and defined the new GO Slim class to store and manage GO Slim annotations for gene products.

### Cell and tissue-specific gene expression

We designed three new classes Expression, Tissue and Microarray platform to store and manage the cell and tissue-specific gene expression data from the Barcode database (29). The Barcode algorithm transforms the raw expression values into normalized expression scores between 0 and 1 that reflect the proportion of the specific source cell/tissue samples (corresponding to the same specific microarray platform) where a gene was judged to be expressed. A score of 0 implies that the gene was not ‘sufficiently’ expressed (29) in any samples of a given tissue, whereas a score of 1 corresponds to ‘sufficient’ expression in all the examined samples. For instance, a Barcode score of 0.25 for a gene in a tissue (e.g. liver) indicates that the gene was judged to be ‘sufficiently’ expressed in 25% of the liver samples where the expression of this gene was measured. Genes were judged to be ‘reliably’ expressed in specific cell/tissue types if they were associated with a Barcode score of ≥0.5 (i.e. a gene was ‘sufficiently’ expressed in ≥50% of the processed cell/tissue samples).

### TargetMine auxiliary toolkit

The new auxiliary toolkit was based on Google Web Toolkit, an open-source development toolkit for building and optimizing complex browser-based applications, using Java as the development language. The auxiliary toolkit processes the input query and retrieves the relevant data using the InterMine Java API. The results are displayed in the form of interactive graphs and tables.

#### Biological enrichment analysis

The enrichment of biological themes associated with the query list was assessed by Fischer’s exact test and the inferred *P* values were further adjusted for multiple test corrections to control the false discovery rate using the Bonferroni, Benjamini and Hochberg or Holm–Bonferroni procedures (66–68). By default, the significantly enriched biological themes (that satisfied a condition of *P* ≤ 0.05 after a multiple test correction with the Benjamini and Hochberg procedure) are summarized for visualization. Genes associated with enriched KEGG pathways were mapped and highlighted on the corresponding KEGG pathway map using the KEGG mapper ‘search pathway’ tool.

The association heatmap was implemented using Data-Driven Documents (D3.js), an open source JavaScript library for manipulating documents based on data (http://http://d3js.org/). We employed a host of in-house scripts to transform the enriched biological theme associations into a customisable heat map of gene–biological theme associations. First, the selected gene–biological theme associations were transformed into a binary index; to simplify the computation, all biological themes that were associated with a gene were assigned the value of 1 and the rest were assigned the value of 0, as indicated in a colour bar aside the heatmap. The collective binary indices were then collated into a matrix where each row is defined as the functional association profile of each gene. Next, Euclidean distances were computed for each pair of gene-association profiles and average-linkage hierarchical clustering was performed with the resulting distance metric. The output was then organized into a matrix and plotted as a grid of squares.

#### Composite interaction network

The Cytoscape.js plugin was employed for the visualization of the composite interaction networks, which were generated using a series of in-house scripts to retrieve the different types of interaction data within TargetMine and then merge them into a singular network. For better reliability, only the high-confidence direct physical PPIs were included within the composite interaction network.

## Supplementary data

Supplementary data are available at *Database* Online.

Supplementary Data
