# Supplementary material for: An integrative data analysis platform for gene set analysis and knowledge discovery in a data warehouse framework
Source: Database (Oxford). 2016 Mar 17;2016:baw009. doi: 10.1093/database/baw009 (PMC4795931; doi:10.1093/database/baw009)
Supplement: Supplementary Data [file supp_2016_baw009_index.html]

An integrative data analysis platform for gene set analysis and knowledge discovery in a data warehouse framework — Supplementary Data 

# An integrative data analysis platform for gene set analysis and knowledge discovery in a data warehouse framework

## Supplementary Data

files

- Supplementary Data - xlsx file
